# Optimization of drying parameters and texture properties of winter jujube slices by radio frequency combined with hot air

**DOI:** 10.3389/fnut.2024.1523078

**Published:** 2025-01-07

**Authors:** Yang Li, Chenyan Yang, Shuaitao Cao, Ruijie Guan, Bowen Zhang, Xuedong Yao, Qiang Wang, Wancheng Dong, Yong Huang

**Affiliations:** ^1^College of Mechanical and Electrical Engineering, Shihezi University, Shihezi, China; ^2^Key Laboratory of Northwest Agricultural Equipment, Ministry of Agriculture and Rural Affairs, Shihezi, China; ^3^Key Laboratory of Modern Agricultural Machinery Corps, Shihezi, China

**Keywords:** winter jujube slices, drying, texture properties, microstructure, comprehensive balance method, matrix analysis method

## Abstract

In order to improve the drying quality of winter jujube slices and find the best drying process parameters, RF + HA (radio frequency combined hot air) drying technology was used in this study to study the effects of plate spacing, RF application time, and RF interval time on the quality of winter jujube slices. Vitamin C (*VC*) content, red and green value (a*), and drying rate (*DR*) were used as quality indexes, and the changing trend of texture properties was analyzed. According to the conclusion of the single-factor experiment, the orthogonal experiment is carried out, and the parameters of each factor in the orthogonal experiment are optimized by the comprehensive balance method and matrix analysis method. The results showed as follows: (1) Plate spacing, RF application, and interval time all significantly affected the drying properties in the single-factor test (*p* < 0.05). The *VC* content of winter jujube slices increased and then decreased with the increase in the three factors. (2) In the orthogonal test, the order of influence of each factor on the quality of the winter jujube tablet is plate spacing > RF interval time > RF application time. The optimum RF heat treatment parameters are plate spacing of 100 mm, RF application time of 3 min, and RF interval time of 2 min. Under these conditions, the *VC* content of the winter jujube slices was 258.35 mg/100 g, a* was −9.47 and the *DR* was 0.64 g/min. (3) RF + HA has more advantages in shortening drying time and maintaining shape, reducing hardness by 12.6 ~ 18.7% and crispiness by 13.8 ~ 20.4%, the microstructure of jujube slices shows a regular honeycomb shape. The research results provide a new drying combination mechanism and process optimization scheme for improving the drying technology of winter jujube slices in industrial production.

## 1 Introduction

Winter jujube (*Ziziphus jujuba* Mill. cv. Dongzao) is rich in various vitamins, amino acids, sugars, flavonoids, alkaloids, cyclic nucleotides, and other nutrients and trace elements such as potassium, calcium, phosphorus, and iron (1). It is an essential leisure and medicinal food in daily life. In addition, winter jujube has the effects of delaying aging, anti-oxidation, promoting blood circulation, improving immunity, anti-cancer cancer, and anti-fatigue (2). Although winter jujube can be eaten directly, it is not conducive to storage, the market usually adopts the method of sliced dry to improve the shelf life of winter jujube, sliced dry is also easy to manufacture, package, transport, and further process. Ma (3) through the different maturity of the winter jujube tissue slice image gray, median filtering, histogram equalization, and binarization pretreatment, found that the slice features have a suitable discrimination performance, and the fractal dimension decreases with the increase in water content. Tepe (4) found that the drying speed of sliced jujube is accelerated with the increase in drying temperature and water-soluble vitamins were significantly reduced during drying. Bao (5) after short-wave pretreatment of fresh jujube slices, reduced the loss of reducing sugar, increased the total soluble solids, and decreased the Browning index. All the above studies showed that winter jujube slicing can improve the drying rate (*DR*) and drying quality of winter jujube.

At present, there are many methods and related research reports on jujube slice drying at home and abroad. The commonly used methods include hot air drying (HAD) (6), gas jet impact drying (GJPD) (7), vacuum freeze drying (VFD) (8), vacuum pulsation drying (VPD) (9), etc. HAD has low input and maintenance costs and simple drying technology. So, it is still one (10, 11) of the most widely used drying methods in the world, but there are problems with extended drying time and reduced storage quality. At the beginning of drying, the external heating of the material to remove the surface-free water is more efficient. At the same time, removing the free water, jujube slices lose their biological activity due to the loss of too much water. In the later drying period, the removal of bound water requires more energy, and the “hard shell” phenomenon is prone to appear on the outer skin, which obstructs the escape and evaporation of internal water, affects the drying speed in the later period, and leads to the texture of jujube slices to fission (12). Therefore, different stages of HAD are divided. It can better observe and analyze the texture properties and internal material content changes of winter jujube slices under different moisture content. Cao (13) analyzed the color changes of jujube slices at different temperatures and found that color difference changed significantly with the increase in temperature in the early period of HAD but changed very slowly in the later periods of drying. Qin (14) found that the drying stage had a significant impact on the released volatile compounds when analyzing the aroma profile of mushrooms in different HAD stages, and the concentration of ketones and alcohols in the early drying stage decreased the fastest. In the pre-and middle drying stages, the internal microstructure of the material was less damaged, the moisture retention was significant, and the surface color change was small. In the last 10–15% of drying, 50% of the whole drying stage was consumed, and too long drying time would affect drying quality and drying effect. However, HAD alone can no longer solve the problems of poor texture properties and serious internal structure damage after drying.

Radio frequency (RF) is a volumetric heating method that can generate heat quickly and deeply in the substrates of food. It is an electromagnetic wave in the frequency range of 3 kHz–300 MHz, which can penetrate food and interact with ions, atoms, and molecules to generate internal heat. It is a new heating technology with high thermal efficiency, low energy consumption, and an adjustable heating rate, which is part of the whole heating (15). RF heat treatment is widely used, currently mainly used in food drying (16), sterilization (17), and insecticide fields (18), among which RF + HA drying in the field of food drying is more and more widely used. Shewale (19) compared RF + HA (radio frequency combined hot air) drying and single HAD and found that RF + HA drying time was shortened by 37%, energy consumption was reduced by 52%, and the application of RF in the secondary drying stage with low air humidity accelerated the drying process to retain the quality attributes of the product better. Gong (20) applied RF and treatment and found that the RF-assisted heat treatment required less time than the single HAD. It can be seen that the reports of RF + HA drying mainly focus on the research of heating uniformity and quality change, but there are few reports on the optimization of parameters of the application mechanism under RF heat treatment.

The texture property of food refers to a set of physical properties felt through contact, which, together with the appearance, flavor, and nutrition of food, constitute the four major quality elements of food. In the drying process, the change in moisture content affects the texture properties and shrinkage properties of fruits and vegetables, which plays a vital role in measuring product quality. Texture properties are critical quality attributes of jujube slices, which have a more significant impact on consumers’ taste and acceptance of products. For example, crispiness, hardness, and chewiness are typical parameters reflecting the quality structure of fruits and vegetables. Low crispiness and low hardness can reflect the difficulty of chewing in the process of edibleing (21). Xia (22) analyzed the influence of jujube kernel powder added to jujube powder on its texture properties, and the addition of jujube kernel powder can effectively reduce the color difference change of jujube powder in the moisture absorption process and reduce the hardness and viscosity of jujube powder in the agglomeration process. Adding jujube kernel powder to jujube powder can effectively reduce the water content of jujube powder, thus significantly reducing the caking degree of jujube powder.

In view of this, the study took Xinjiang winter jujube as the test material, Vitamin C (*VC*) content, red and green value (a*), and drying rate (*DR*) as the quality indexes, explored the influences of plate spacing, RF application time, and RF interval time on the above indexes and their variation rules, analyzed the influences on the texture properties parameters, and obtained the parameter combination optimization scheme of RF heat treatment application mechanism under HAD conditions. The suitable conditions of RF + HA drying intervention were determined to provide theoretical and data references for the optimization of the fruit and vegetable drying process.

## 2 Materials and methods

### 2.1 Sample preparation

The experimental materials were winter jujube (longitudinal diameter (29 ± 2) mm, transverse diameter (24 ± 2) mm), produced in Aksu, Xinjiang, purchased from Shihezi Farmers Market. Winter jujube with bright red color, complete appearance and, consistent maturity were selected, cleaned, dried and, cored into (10 ± 1) mm thick round jujube slice samples and placed in a refrigerator at constant temperature (4 ± 1) °C, and constant humidity (relative humidity 96 ± 2%) for 24 h for use. Before the drying test, the initial moisture content of jujube slices measured by oven method (23) is 79.2 ± 1% w.b. (wet basis).

### 2.2 Instruments and equipment

**Figure 1:**
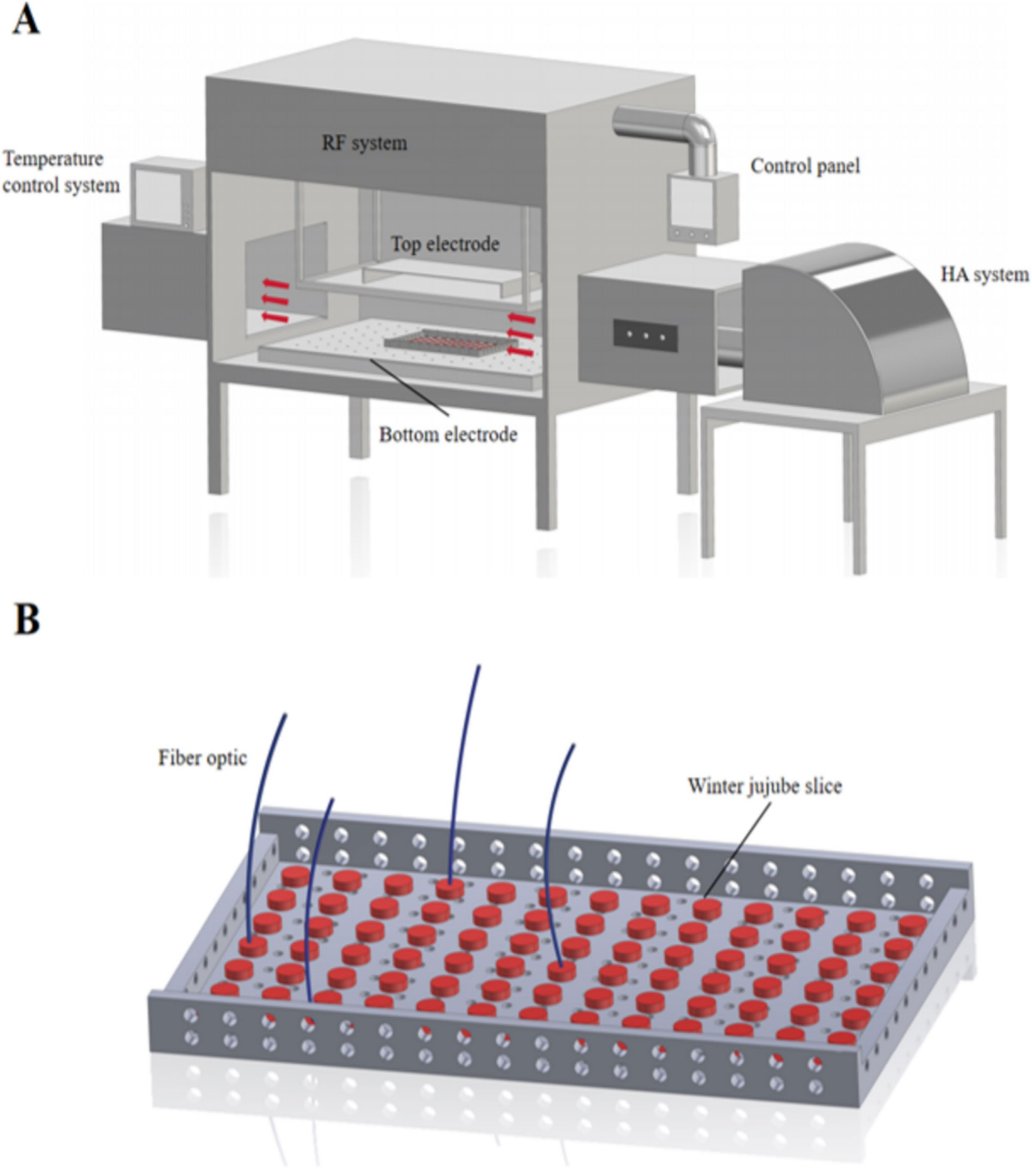
Structure diagram of hot air and radio frequency combined drying device. **(A)** RF drying system; **(B)** material tray and optical fiber temperature measurement positions.

The drying device used in the test is a COMBI6-S hot air and RF combined drying system (SO6B, Stray Field International Limited, Wokingham, United Kingdom), with a power of 6 kW and a frequency of 27.12 MHz (Figure 1A). The area of the RF heating field composed of the plate is 750 × 550 mm, and the spacing of the plate is adjustable from 80 to 200 mm. The temperature of the hot air system is adjustable from 25 to 100°C, and the wind speed is adjustable from 0 to 1 m/s. The hot air generated during the drying process is discharged through the vent hole of the lower plate and transmitted to the drying chamber above the lower plate to dry the material. Before the test, the jujube slices were evenly laid on the rectangular mesh material plate. The material plate made of polypropylene with a small dielectric loss factor was used in the drying device, the size of which was 350 × 250 × 10 mm. The internal temperature change during the drying process was observed in real-time by using a fiber optic thermometer (Q-FTS-D1F00, Optoelectronic Technology Co., LTD., Xi’ an, China). The jujube slices of 1,000 g were weighed and evenly spread in the mesh material disc, and the optical fiber temperature sensor was inserted into the center of the jujube slice (Figure 1B). Jujube slices were taken out and weighed every 30 min, and a random sample was taken from the placed pellet to detect the corresponding index.

Other test equipment include electronic balance (BSM220.4, Zhuojing Electronic Technology Co., LTD., Shanghai, China); Freezer (BCD-267G, Hisense Rongsheng Freezer Co., LTD., Guangdong, China); color-difference meter (CHROMA METER CR-410, Konica Minolta Holdings, Inc., Osaka, Japan); portable photosynthesis measurement system (Li-6400, Li-COR Corporation, Nebraska, United States); critical point dryer (HCP-2, Hitachi Ltd., Tokyo, Japan); scanning electron microscope (JSM-6490LV, Japan Electronics Co., LTD, Tokyo, Japan); and texture analyzer (TA-XT2; Stable Micro System Ltd., Leicestershire, United Kingdom); infrared camera (Fluke RSE600, Fluke Electronics Instrument Co., Ltd., Washington, United States).

### 2.3 Experimental methods

#### 2.3.1 HAD stage division

When Zang (24) analyzed the microstructure of dried jujube slices by RF + HA, they defined 2–4 h as the middle drying period of combined drying by RF + HA. At this time, the *DR* of jujube slices was significantly higher than that of single hot air. The microstructural gap size of jujube slices was regular, the shape was similar, and the arrangement was porous, showing a relatively regular honeycomb shape, and the micro skeleton was less wrinkled. Yao (16) used RF + HA to dry the jujube slices in different drying stages, namely, the early stage (0–2 h), the middle stage (2–4 h), and the late stage (4–6 h). Compared with the HAD group, the early stage group reduced the number of cells with roundness of less than 0.4 by 5% and had the best effect in the experimental group. In addition, the middle stage group showed better results than the other groups, with 18.6 percent fewer cells with a cross-sectional area of less than 200 μm and 48.8% fewer cells with a circumference of less than 25 μm. The total color difference was the smallest, and the ascorbic acid retention was the greatest. Therefore, in the drying industry, it is feasible to improve the drying quality of agricultural products and reduce energy consumption by adopting the phase-controlled radio-frequency assisted HAD method. In this test, 0–2 h is divided into the early drying stage, 2–4 h is the middle drying stage, 4–6 h is the late drying stage, and RF heat treatment was applied during the middle drying stage.

#### 2.3.2 Single factor test method

According to the pre-test and related scholar’s (25) research, plate spacing has a significant impact on the heating rate, but too fast temperature rise will lead to rapid degradation of *VC* and brown color, and relevant studies generally use 90 ~ 180 mm. Too long RF application time will cause the water in the material to migrate too fast, affecting the drying quality and experimental results. The RF interval time should not be too long, otherwise the RF application will not have a significant impact on the experimental results. Therefore, this study determined the factor level of the single factor test and set different plate spacing (100, 110, 120, 130 mm), RF application time (1, 2, 3, 4 min), and RF interval time (1, 2, 3, 4 min) respectively to carry out the drying test. At every 60-min interval, samples were taken out to measure the moisture content of jujube slices. When the moisture content (w.b.) of the jujube slices was lower than 7%, the test was ended, and the test was repeated 3 times for each group, and the average value was taken (Table 1).

**Table 1:** Experiment design and experimental parameters.

Series	Plate spacing/mm	RF application time/min	RF interval time/min
1	100	1	1
2	110	1	1
3	120	1	1
4	130	1	1
5	100	1	1
6	100	2	1
7	100	3	1
8	100	4	1
9	100	3	1
10	100	3	2
11	100	3	3
12	100	3	4

**Figure 2:**
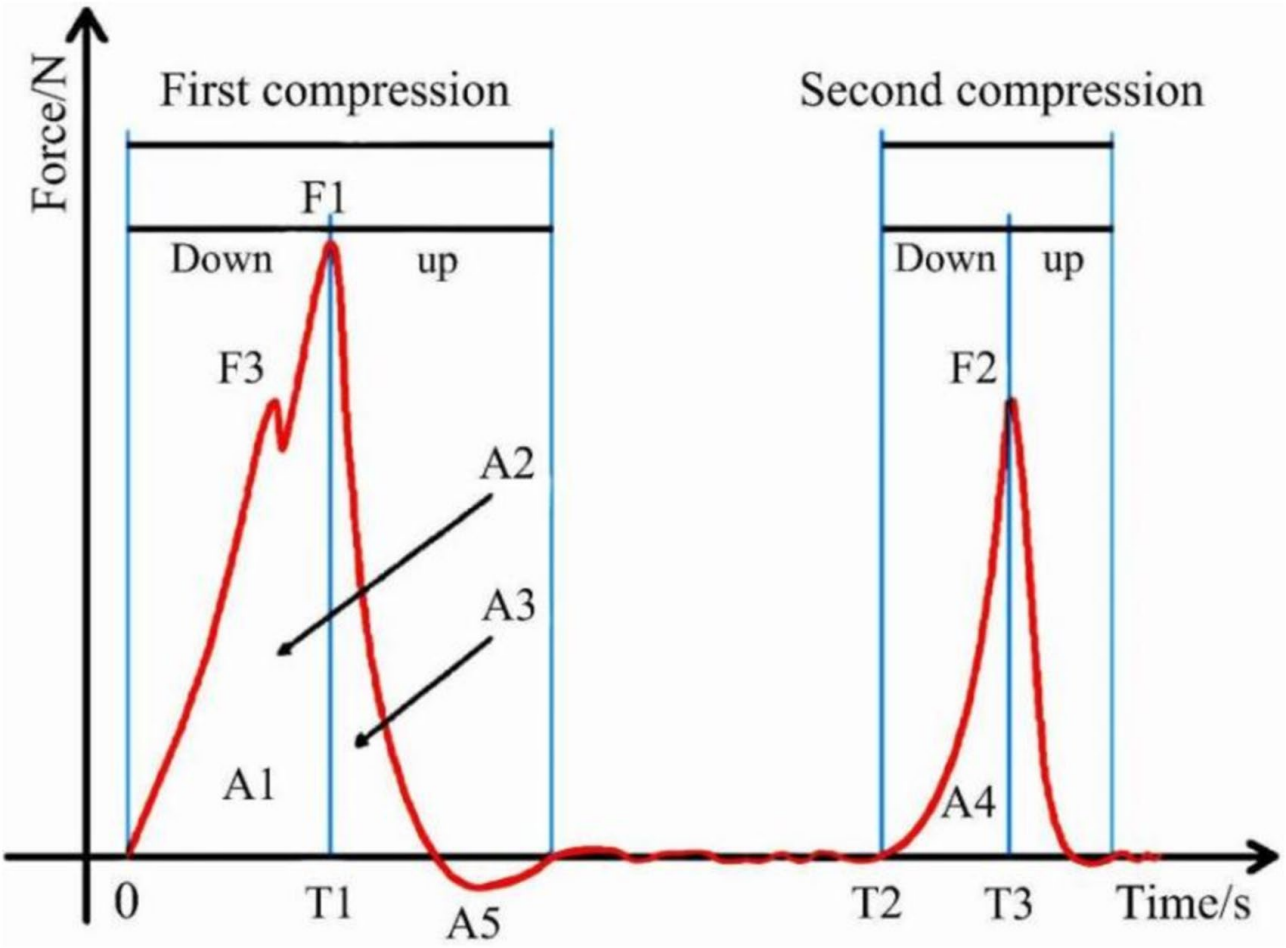
Structural properties TPA curve analysis diagram.

In order to make the HAD the jujube slices more fully and dry the diffused water vapor in the RF heat treatment process, it is necessary to set up ventilation holes on the lower plate and the material tray. When the jujube slices are too thick, the hot air makes it difficult to take away the internal moisture of the jujube slices, resulting in uneven drying inside and outside, affecting the drying quality. Therefore, according to the size of the jujube slice thickness of 10 ± 0.5 mm, to ensure that the RF energy can penetrate the material under the condition of the test plate spacing and ensure that the hot air takes away the water vapor from the outside of the jujube slices. It will not cause uneven drying or burning due to heat accumulation. Fruit and vegetable slices in the drying process of the edge part will lose water deformation, and the edge of the slice will shrink and bulge, making the RF field concentrated on the edge of the slice and causing an “ignition” phenomenon (26), to avoid the edge of the jujube slice contact with each other and form overheating or ignition phenomenon, it is necessary to leave a gap when placed in each jujube slice. Place the material tray loaded with winter jujube slices in the center of the lower plate (Figure 2).

Liu (27) put the jujube slices into the frozen infrared drying chamber and set the infrared heating temperature to 50 ~ 80°C. He found that the drying time would decrease with the increase in temperature, and *VC* retention would increase first and then decrease. When the drying temperature is too low, the drying time will be extended, and the drying quality will be low. When the drying temperature is too high, resulting in excessive water loss, jujube slices drying heat and mass transfer imbalance, and serious shrinkage, dry quality also becomes low. At 50 ~ 65°C, the *VC* retention rate gradually increases with the increase in temperature, which is because the drying time is shortened with the increase in temperature, which is conducive to *VC* retention. When the temperature is higher than 65°C, the *VC* retention rate gradually decreases with the increase in temperature. In the infrared HAD, considering the heat dissipation of the infrared plate, the hot air temperature should not be very high (28). Therefore, the hot air temperature in this test is fixed at 65°C, and the wind speed is 0.5 m/s.

#### 2.3.3 Orthogonal test method

According to the results of the prior experiment and single factor test, under the condition of frequency, 27.12 MHz and output power of 6 kW, three optimal levels of plate spacing (100, 110, 120 mm), RF application time (2, 3, 4 min) and RF interval time (2, 3 and 4 min) were selected. The *VC* content, a*, and *DR* were tested by three-factor and three-level orthogonal test to determine the best RF process conditions. The orthogonal table L9 (3^3^) was selected (Table 2).

**Table 2:** Orthogonal test factors and level table of RF heat treatment on HAD jujube slices process.

	A	B	C	D
Levels	Plate spacing/mm	RF application time/min	RF interval time/min	Blank column
1	100	2	2	1
2	110	3	3	2
3	120	4	4	3

#### 2.3.4 Vitamin C content

The *VC* content was determined by the 2, 6-dichloro-indophenol titration method according to the Chinese standard GB 5009.86-2016 (29). The specific methods are as follows:

*Test solution preparation:* Put the test sample in the crusher (accurate to 0.001 g), add 100 g oxalic acid solution, and quickly break, shake well for reserve.
*Titration:* Add 10 mL filtrate into 50 mL conical bottle, titrate with calibrated 2, 6-dichloro-indophenol solution until the solution is pink for 15 s without fading, and add blank test at the same time.

#### 2.3.5 Determination of a*

According to the Chinese standard GB/T 32714-2016 (30) of winter jujube, the best grade is ochre red, and Wang et al. (31) found in the influence of maturity on vacuum pulsating drying kinetics and product quality that the brightness and blue and yellow values of winter jujube fruits will decrease with fruit ripening, and the a* will increase and have a “redturn” phenomenon. Cao et al. (32) fitted the color changes of winter jujube slices under different drying temperatures, and found that the a* value showed a significant change in the early drying stage, the b* value increased with the extension of drying time, and the change was not significant in the early and middle drying period. Therefore, a* is selected as the quality index as the color judgment index of the RF stage. Select one of the jujube slices in each group and use a color-difference meter to determine its a*. Repeat the parallel test 3 times to take the final average value as the test data. The lager the a* is, the closer the color is to pure red.

#### 2.3.6 Drying characteristics analysis

The wet basis moisture content of jujube slices at moment *t* was calculated as follows formula 1 (33).


(1) $$M_{t}=\frac{W_{t}-W_{0}}{W_{0}}$$

where *M_t_* is the wet basis water content, g/g; *W_t_* is the t moment of the total mass of the material, g; *t* is the drying time, min; and *W_0_* is the dry material mass, g.

The drying rate (*DR*) in the drying process of jujube slices is calculated according to formula 2:


(2) $$DR=\frac{M_{t}-M_{t+\Delta t}}{\Delta t}$$

where *DR* is drying rate, g/min; *M_t_* is material weight at time t, g; *M_t + Δt_* is the weight of material at time *t + Δt*, g; *Δt* is the time when the RF is applied, min.

#### 2.3.7 Texture properties detection method

Texture Analyser can express the physical properties of the sample digitally. Among them, Texture Profile Analysis (TPA) is a compression method that is widely used to determine the texture of food. The principle is to simulate the process of food chewing, and the sample is compressed twice to determine the pressure of the probe on the sample and other related texture parameters.

In this study, the TPA of jujube slices under different drying conditions was carried out with a texture analyzer, and the texture analysis method of Wang (31) was modified. The test parameters were set as follows: the P/50 probe was used, the pre-test speed was 1 mm/s, the test speed was 0.5 mm/s, and the upward speed was 1 mm/s after the test so that the compression deformation of the jujube slices could reach 50%, and the compression pause time was 5 s twice. Each group of samples was measured 10 times, and the average value of the results was taken (Table 3).

**Table 3:** Definition indexes of texture properties of jujube slices.

Texture parameter	Definition
Crispness	The first obvious peak F3 during the first downdraft
Hardness	Maximum peak value F1 during the first down
Elasticity	The ratio of the time experienced when the second downward pressure reaches the peak (T3-T2) to the time T1 experienced when the first downward pressure reaches the peak
Cohesion	The ratio of the peak area A4 from the second compression to the peak area AI from the first compression
Masticatory	Product of hardness, cohesion, and elasticity
Resilience	The ratio of the peak area A3 of elastic energy released by the jujube slices when the probe first goes up to the peak area A2 of probe energy dissipation during compression
Adhesiveness	The negative area A5 between the end of the first compression and the beginning of the second compression

#### 2.3.8 Determination of texture properties and temperature

Open the HAD device, set the hot air temperature, and preheat for 1 h to ensure stable and uniform temperature in the cavity. Open the RF device, set the test parameters, put the material plate between the upper and lower plates, and start the RF heating (choose 2–4 h in the drying process according to the RF processing time of the preliminary test) (Table 4). The jujube slice samples for texture and temperature measurement were removed from the drying device every 30 min, and the temperature of the top surface of the winter jujube slices on the plate was recorded with infrared camera within 20s.

**Table 4:** Design for the experiments.

Methods	Heating time/h	Hot air temperature/°C	Plate spacing/mm	Air velocity/(m·s^−1^)
HA	/	55	/	0.5
		65		
		75		
RF + HA	2	55	110	
		65		
		75		

#### 2.3.9 Acquisition of microscopic images

The winter jujube slices of different periods were divided into 3 × 3 × 4 mm cubes (34), and the samples were placed in 2.5% glutaraldehyde fixing solution for 12 h, then poured out the fixing solution, rinsed with phosphoric acid buffer (pH 7.0) for 3 times, fixed with 1% osmic acid solution for 2 h, rinsed with phosphoric acid buffer (pH 7.0) for 3 times. The jujube slice samples were dehydrated with an ethanol solution of different concentrations (30, 50, 70, 80, 90, 95, 100%) for 20 min. The dehydrated samples were dried in the HCP-2 critical point dryer. After gold spraying, the dried samples were placed under a JSM-6490LV electron scanning microscope to observe the cross-section microstructure. The working voltage was set to 4.0 kV, and the magnification was 100 times to obtain the microscopic images of red jujube slices.

#### 2.3.10 Multi-index result analysis method

1. The comprehensive balance method is to analyze the orthogonal test data of multiple indicators, analyze each indicator one by one, find out the optimal combination of its factor levels, and then conduct a comprehensive balance according to the importance of each indicator, primary and secondary factors and the pros and cons of the levels derived from each indicator, and finally determine the overall optimal combination of factor levels (35).
2. The matrix analysis method analyzes the orthogonal test data of multiple indicators: if there are m factors in the orthogonal test process, each factor has n levels;

Definition 1: Examine the index layer matrix *M*, the lager the index is, the better,

If the average of the *i* factor *j* level is kij, then *Kij* = *kij*; The smaller, the better, then let *Kij* = 1/*kij*, establish matrix (Equation 7).

Definition 2: Factor layer matrix *F*, The matrix of (Equation 8) is established according to Equation (3).


(3) $$F_{i}=1/\overset{n}{\underset{j=1}{\sum }}K_{ij}$$

Definition 3: Horizontal layer matrix *L*, if the range of factors in the orthogonal test is *Ri*, then let Equation (4) establish matrix (Equation 9).


(4) $$L_{i}=R_{i}/\overset{n}{\underset{i=1}{\sum }}R_{i}$$

Definition 4: The weight matrix of index *E* is *W = MFL*, and the (Equation 10) is established according to Equation (5).


(5) $$W^{T}=\left[w_{1}\;w_{2}\;\ldots \;w_{\mathrm{lm}}\right]$$

The experiment is the orthogonal design of *S* indicators, and the total weight matrix of the indicators is Equation (6).


(6) $$Y=\overset{s}{\underset{i=1}{\sum }}\frac{E_{i}}{F}/S$$

(7) $$M=\left[\begin{array}{cccc} K_{11} & 0 & 0 & \begin{array}{cc} \ldots & 0 \end{array}\\ K_{12} & 0 & 0 & \begin{array}{cc} \ldots & 0 \end{array}\\ \ldots & \ldots & \ldots & \begin{array}{cc} \ldots & \ldots \end{array}\\ \begin{array}{c} K_{1m}\\ 0\\ 0\\ \begin{array}{c} \ldots \\ 0\\ \ldots \\ \begin{array}{c} 0\\ \begin{array}{c} 0\\ \ldots \\ 0 \end{array} \end{array} \end{array} \end{array} & \begin{array}{c} 0\\ K_{21}\\ \begin{array}{c} K_{22}\\ \ldots \\ K_{2m}\\ \ldots \end{array}\\ \begin{array}{c} \begin{array}{c} 0\\ 0\\ \ldots \end{array}\\ 0 \end{array} \end{array} & \begin{array}{c} 0\\ \begin{array}{c} 0\\ 0\\ \ldots \\ 0 \end{array}\\ \ldots \\ \begin{array}{c} \begin{array}{c} 0\\ 0\\ \ldots \end{array}\\ 0 \end{array} \end{array} & \begin{array}{c} \begin{array}{c} \begin{array}{cc} \ldots & 0 \end{array}\\ \begin{array}{cc} \ldots & 0 \end{array}\\ \begin{array}{cc} \ldots & 0 \end{array}\\ \begin{array}{cc} \ldots & \ldots \end{array} \end{array}\\ \begin{array}{cc} \ldots & 0 \end{array}\\ \begin{array}{cc} \ldots & \ldots \end{array}\\ \begin{array}{c} \begin{array}{cc} \begin{array}{c} \ldots \\ \ldots \\ \ldots \end{array} & \begin{array}{c} K_{n1}\\ K_{n2}\\ \ldots \end{array} \end{array}\\ \begin{array}{cc} \ldots & K_{\mathrm{nm}} \end{array} \end{array} \end{array} \end{array}\right]$$

(8) $$F=\left[\begin{array}{cccc} F_{1} & 0 & 0 & 0\\ 0 & F_{2} & 0 & 0\\ \ldots & \ldots & \ldots & \ldots \\ 0 & 0 & 0 & F_{4} \end{array}\right]$$

(9) $$L=\left[\begin{array}{c} L_{1}\\ L_{2}\\ \ldots \\ L_{n} \end{array}\right]$$

(10) $$E=\left[\begin{array}{c} E_{1}\\ E_{2}\\ \ldots \\ E_{m} \end{array}\right]$$

The multi-index analysis method can calculate the weight matrix (35) of the influence of each factor and level on the test investigation index, and get the primary and secondary order of the influencing factors and the optimal parameter combination.

#### 2.3.11 Statistical analysis

In the determination of *VC* content, a* and *DR*, three parallel sets were set in each group. The average values were used for data processing and statistical analysis by orthogonal design assistant V3.1, Origin2022, and Excel2010 software.

## 3 Results and discussion

### 3.1 Analysis of results of single factor experiment

#### 3.1.1 Influence of different test conditions on *VC* content of HAD jujube slices

**Figure 3:**
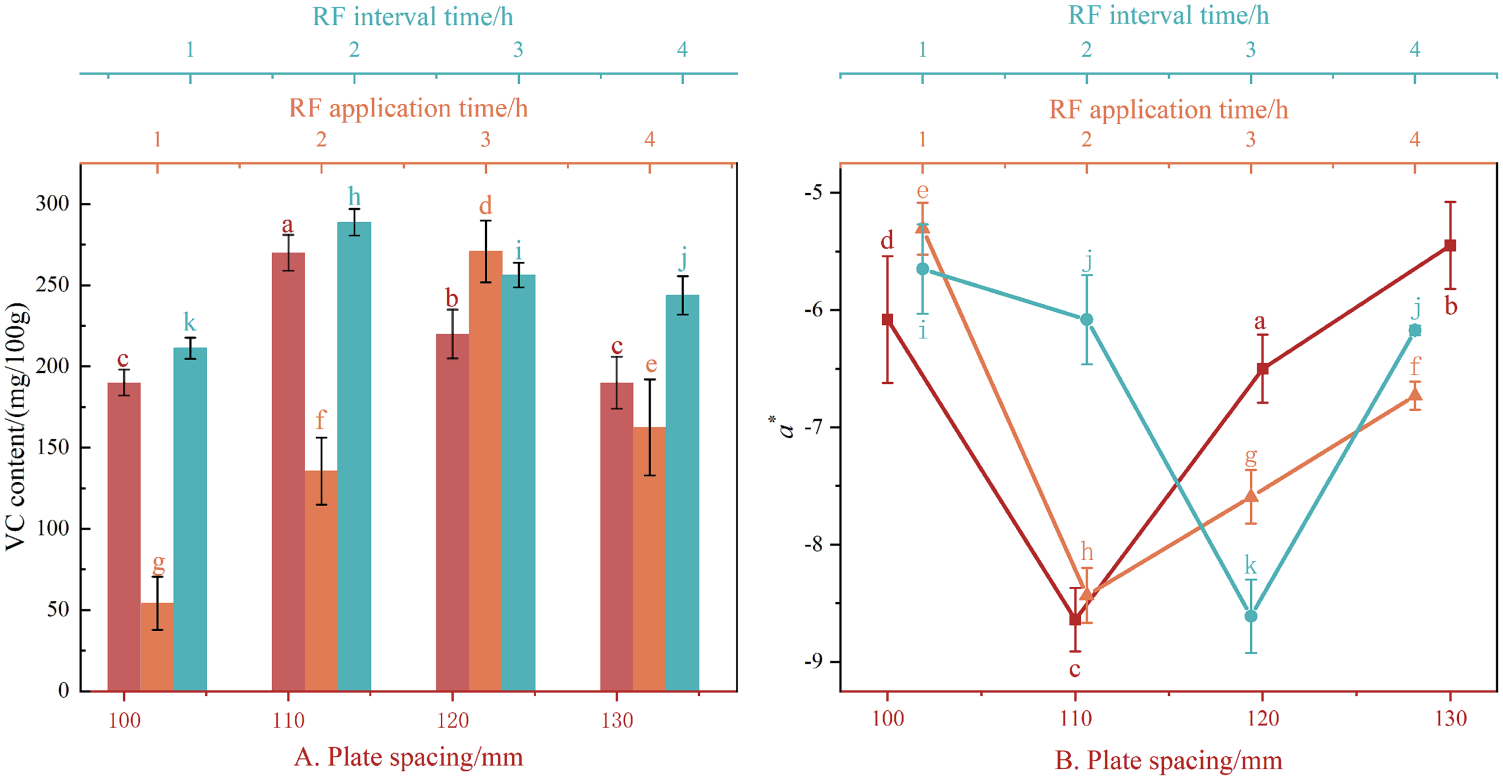
Effect of different test conditions on *VC* content **(A)** and a* **(B)** of hot air dried jujube slices. Different letters in the figure reveal significant differences (*p* < 0.05). a. RF application time is 2 min and RF interval time is 2 min; b. Plate spacing is 110 mm and RF interval time is 2 min; c. Plate spacing is 110 mm and RF application time is 2 min. Under the same single-factor test conditions, different lowercase letters in the same column indicate significant differences.

Under the single-factor condition of plate spacing (Figure 3), when plate spacing is 110 mm, *VC* content is the best (0.27 g/100 g). With the increase in plate spacing, the *VC* content in the jujube slices presents a trend of first increasing and then decreasing because changing the plate spacing changes the power of RF heat treatment; the more electromagnetic energy provided by the drying process, the faster the internal temperature of the material rises. When the plate spacing increases, the internal temperature of the jujube slices can be effectively prevented from rising too fast, and the rate of degradation of *VC* nutrients can be slowed down. When the plate spacing is too significant, the energy provided by RF heat treatment is insufficient, resulting in insufficient drying temperature and prolonged drying time, which is not conducive to the retention of *VC*. This is consistent with the measured value of *VC* of *Lycium barbarum* reported by Xu (36). *VC* content will first rise and then fall with the increase in plate spacing because ascorbic acid is a sensitive substance and is easy to oxidize and degrade under high temperatures and aerobic environments. Under the condition of RF application time as a single factor (Figure 3). RF application time is one of the crucial indicators for controlling drying temperature. With the extension of RF application time, the *VC* content of jujube slices presents a trend of first increasing and then decreasing, and the best *VC* retention rate appears when the RF application time is 3 min, which is 0.280 g/100 g. The longer RF application time, the more electromagnetic waves are absorbed by the material, the faster the temperature rises, the drying temperature will increase, and the drying temperature will be too high, which will lead to the quality of jujube slices being reduced; RF application time is too little, the internal temperature of the material and the outside easy to form a significant temperature difference, the drying process will appear on the surface of the material hardening crust phenomenon. Under the condition of single-factor RF interval time (Figure 3). With the extension of the RF interval time, the *VC* content of the jujube slice presents a trend of first increasing and then decreasing, and the *VC* retention rate of the jujube slice is the best, appearing at the RF interval time of 2 min, which is 0.266 g/100 g. Because *VC* is affected by heat sensitivity when heating, too long heating causes the loss of *VC*, and ascorbic acid is easy to decompose at high temperatures, the RF interval time is set in the drying process to avoid the RF heat treatment application time being too long leading to high temperature, affecting the HAD effect.

Under the single-factor condition of plate spacing (Figure 3). With the increase in plate spacing, a* presents a trend of decreasing first and then increasing. The optimal a* is −8.64 when the plate spacing is 110 mm, and the lager a* is, the redder the jujube piece, while the lager plate spacing is not conducive to the maintenance of color. Wang (37) found in the influence of pretreatment on the RF + HA drying process and quality of carrot slices that the input power of the RF field would increase with the decrease in plate spacing and the *DR* of slices, but too low plate spacing would cause an imbalance between the evaporation potential required by the drying water of slices and the input power, which was not conducive to the requirement of temperature control and color protection of dry products. The heating uniformity of the slices has an effect on the color difference value of the slices, and the uneven heating of the slices during the drying process will produce a significant color loss of the slices, which is consistent with the conclusion of the experiment. Under the condition of a single factor of RF application time, with the extension of RF application time, a* shows a trend of decreasing first and then increasing, and the optimal value of a* appears at 2 min (Figure 3). If winter jujube is heated for a long time, the natural pigment in it may be decomposed or destroyed, resulting in the color of winter jujube becoming pale or fading. Long-term heating may cause the color of winter jujubes to darken, which may be due to the reaction of sugars and proteins in the food, resulting in changes in pigments. Under the condition of single factor RF interval time, a* shows a trend of decreasing first and then increasing, and the optimal value of a* appears when the RF interval time is 3 min, which is −9.79, indicating that properly reducing the RF interval time is conducive to the color and quality of winter jujube slices dry products (Figure 3).

#### 3.1.2 Influence of different test conditions on a* of HAD jujube slices

**Figure 4:**
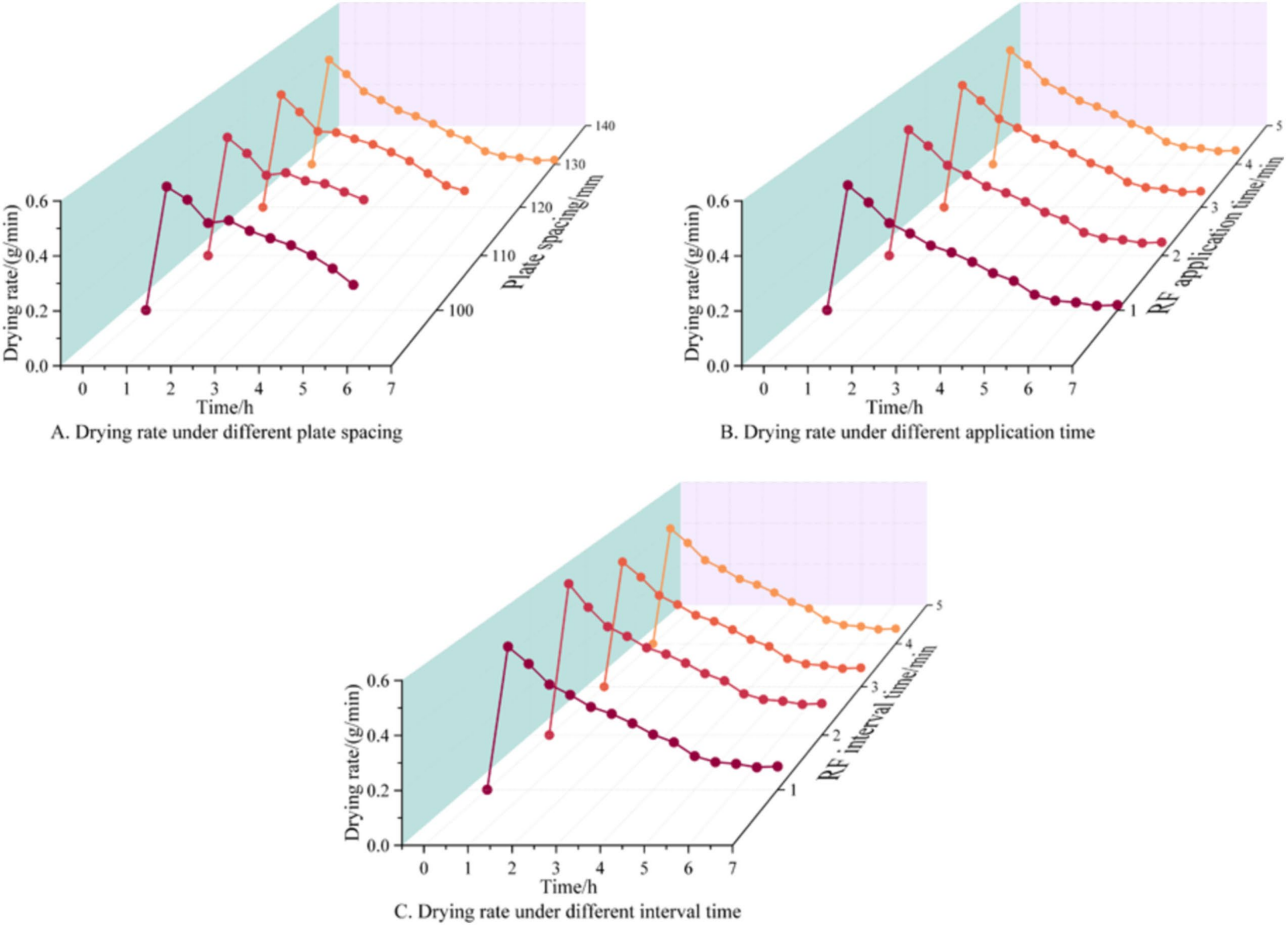
Effect of different test conditions on *DR* of HAD of jujube slices. **(A)** RF application time is 2 min and RF interval time is 2 min; **(B)** Plate spacing is 110 mm and RF interval time is 2 min; **(C)** Plate spacing is 110 mm and RF application time is 2 min.

Under the single-factor condition of plate spacing, the *DR* of the jujube slices presents a trend of sudden rise at first and then decline (Figure 4A). When RF heat treatment is applied to assist HAD at 2 h, the *DR* decreases further. With the increase in plate spacing, the drying speed increased first and then decreased. When the plate spacing was 110 mm, the *DR* of jujube slices reached the maximum, which was 0.35 g/min. This may be the RF heat treatment for volumetric heating, which can achieve the internal temperature rise, make the temperature gradient and humidity gradient in the same direction, and reduce the difficulty of water diffusion. Adjusting the plate spacing will synchronously change the actual power. The power decreases with the increase in plate spacing, the lower the energy of RF heat treatment. When the plate spacing is 110 mm, the *DR* is significantly higher than 100 mm because the distance is close to the jujube slices and absorbs too many RF waves, resulting in serious shrinkage of the surface of the jujube slices and the difficulty of internal water vapor discharge increases. This is similar to the conclusion of the study on the microstructure changes of banana slices during drying by different drying methods (38). Therefore, according to the influence curve of different plate spacing on the *DR* of jujube slices, the suitable plate spacing is 100 ~ 110 mm.

Under the single factor condition of RF application time, with the increase in RF application time, the *DR* curve of jujube slices presents a trend of first increasing and then decreasing, and the *DR* of jujube slices reaches the maximum when the RF application time is 4 min (Figure 4B). This may be because, with the increase in RF application time, the more RF waves are absorbed by the jujube slices, the more heat is converted by the electromagnetic wave, the higher the drying temperature is, and the internal water evaporation is faster. However, too high drying temperature will lead to serious degradation of nutrients, internal water migration to the surface of the material is challenging to evaporate in time, or cause soluble solid condensation on the surface of the material, resulting in a hardening crust phenomenon, and reduce the *DR*. RF heat treatment intervention can improve the internal temperature of the jujube slices, change the direction of heat and mass transfer, and reduce the difficulty of drying, so the longer the RF application time, the higher the drying temperature, the faster the *DR*, but too high drying temperature will also destroy the quality of dry materials, in summary, the commonly used RF intervention range of HAD is 2 ~ 4 min.

Under the single factor condition of RF interval time, different drying interval time had the same drying trend, and the rate curve shows a trend of first increasing and then decreasing (Figure 4C). When the interval time of intervening RF heat treatment is 2 min, the *DR* is the maximum, which is 0.3 g/min. At this time, if the interval time continues to increase, the *DR* will decrease, because with the extension of the interval time, the drying temperature is challenging to maintain, and the *DR* will decrease accordingly. In order to ensure that the drying temperature is maintained within a specific range, the RF interval time should not be too long, but the interval time is too short, which will lead to too fast temperature rise, the internal temperature of the jujube slices is too high, resulting in reduced drying quality, so according to the jujube slices suitable for drying temperature range, determine the RF heat treatment interval time of 2 ~ 3 min.

#### 3.1.3 Influence of different test conditions on texture properties of HAD jujube slices

**Figure 5:**
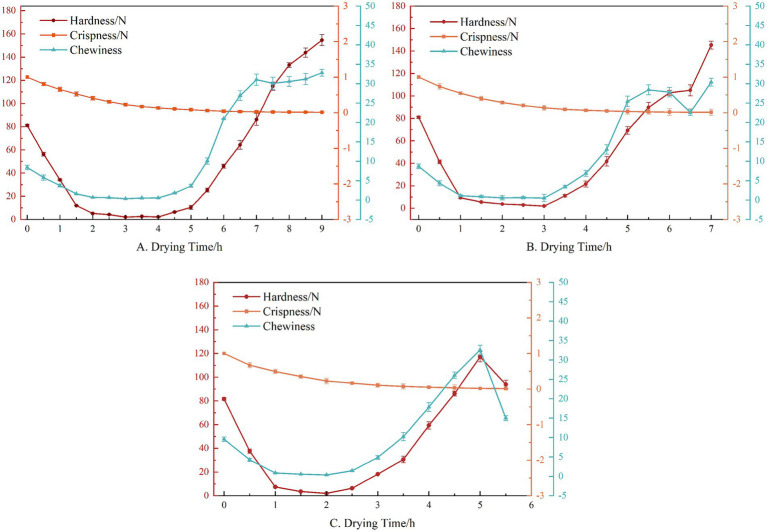
Texture parameters of different drying times. **(A)** The hot air temperature is 55°C; **(B)** The hot air temperature is 65°C; **(C)** The hot air temperature is 75°C.

Crispness, an essential criterion for consumers to select jujube slices (39), is usually expressed by the first prominent pressure peak during the first probe pressing process, and the smaller the peak value, the more brittle the sample. In the drying process, the jujube slices will first undergo softening and then harden the crust. The internal water is challenging to remove, resulting in the rupture of the need for greater force. With the increase of drying temperature, the internal water will gradually diffuse and evaporate so that the crispiness value of jujube slices will gradually reduce. In addition, at a higher drying temperature, the crispiness value of the jujube slices will be lower, and the force required for rupture will also be reduced; that is, the increase in drying temperature and the decrease in moisture content will lead to the decrease in the crispiness of the jujube slices (40). After applying RF heat treatment, a porous structure will be formed inside the jujube slices, which is conducive to the migration and diffusion of internal water and is also conducive to the maintenance of microscopic morphology, making the jujube slices show crispiness earlier, and the crispiness value is lower than that of the single HAD jujube slices. This is similar to Jiang’s research results on microwave drying of okra (41) (Figure 5).

Hardness is another essential parameter in the HAD process, which usually presents changes in two stages. At the beginning of drying, the hardness of jujube slices will gradually decrease, which is caused by the rapid evaporation of surface water. Later, due to the migration and diffusion of internal water, the decline in hardness will slow down. This period is similar to the softening stage of HAD apple slices such as Li (28). When the moisture ratio drops to a certain extent, the jujube slices will enter the hardening stage, and the hardness will increase rapidly. Under different drying temperatures, the hardness of the jujube slices changes in the early and middle stages of drying, which are basically similar, but in the late drying stage, a higher drying temperature will lead to a lower hardness value. In the process of RF heat treatment, the cellulose and pectin of the jujube slices will be degraded, and the water loss will lead to the destruction of the cell wall and cell turgor pressure, resulting in a rapid decline in hardness. Subsequently, due to the change in cell membrane permeability and the increase in cell permeability (20), the internal water diffusion is accelerated, delaying the formation of the surface hard shell, making the jujube slices enter the hardening stage at a lower water ratio (Figure 5).

Chewiness is an essential sensory parameter affecting the drying process, and the changing trend of chewiness and hardness of jujube slices during drying is similar. Under HAD conditions, when the moisture ratio of jujube slices is 10–20%, the chewiness of jujube slices is kept at a low level (0–10), and then the moisture ratio decreases, the epidermis of jujube slices is hardened, the chewiness of jujube slices is increased rapidly (42), and the degree of chewiness of jujube slices is increased significantly. The change in the chewiness range of jujube slices under different drying temperatures is basically similar during drying. When RF heat treatment was applied, the chewiness of jujube slices increased at a lower water ratio (20–30%) (Figure 5).

#### 3.1.4 Influence of different test conditions on temperature of jujube slices

**Figure 6:**
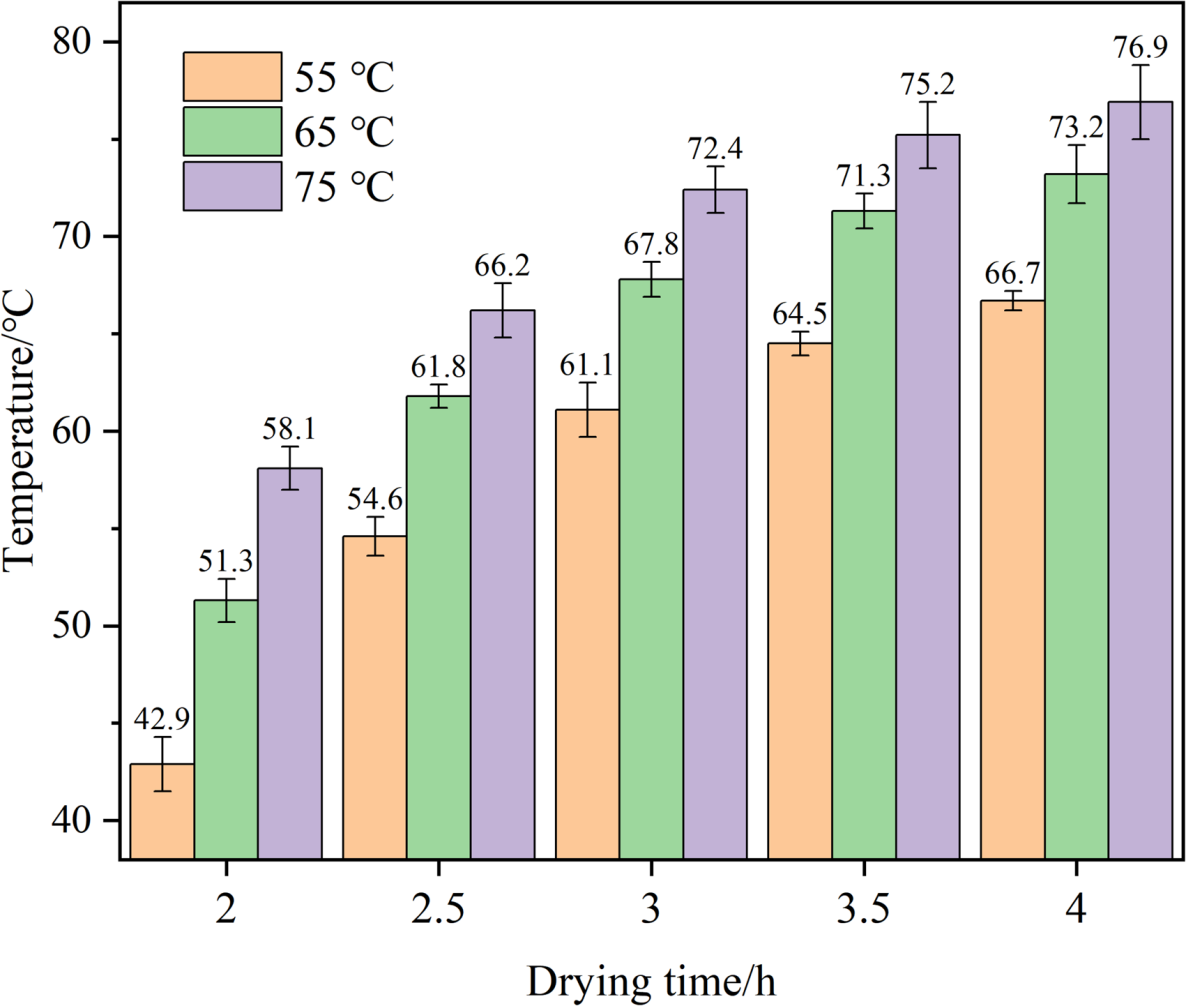
The surface temperature of winter jujube slices at different HAD temperature after applying RF.

In the middle period of HAD (2 to 4 h), when plate spacing is 110 mm, and the RF application time and RF interval time are both 3 min, the surface temperature variation trend (Figure 6) of winter jujube slices treated by RF can be seen that due to the volume heating characteristics of RF, the surface temperature of winter jujube slices in the same period is higher than that of HAD. After adding RF, the overall temperature of winter jujube slices will increase due to the vibration friction of water molecules. Under different hot air temperatures, the average surface temperature of winter jujube slices was significantly increased by RF intervention, and the heating rate showed a slowing trend, which was the same as that of mango slices in the second stage of HAD (43).

#### 3.1.5 Influence of different test conditions on microstructure change of HAD jujube slices

**Figure 7:**
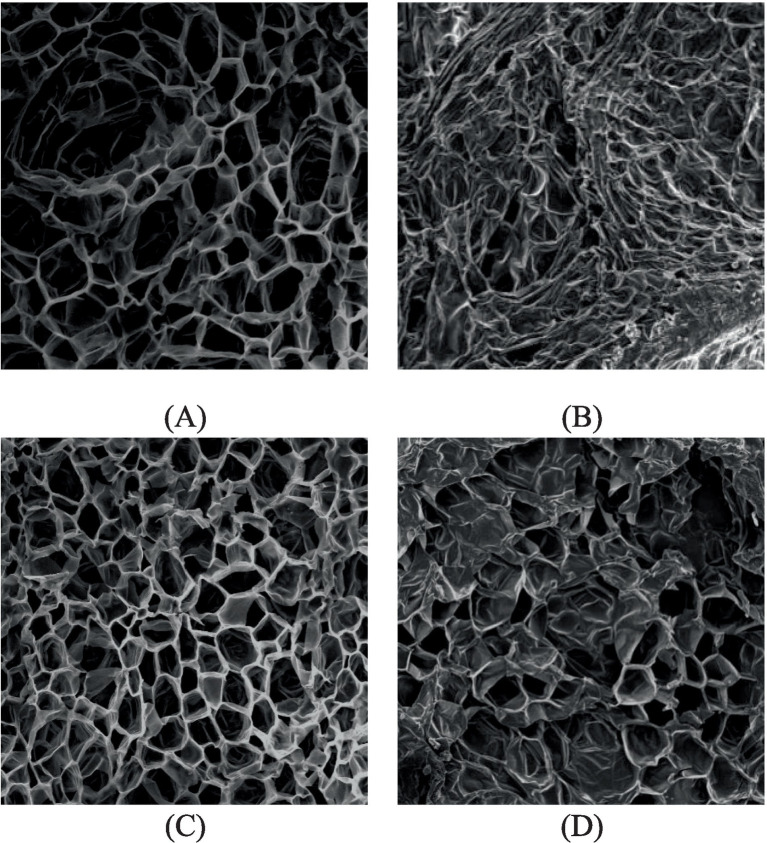
Texture parameters of different drying periods. **(A)** Fresh jujube; **(B)** HAD; **(C)** adding RF heat treatment in the middle stage; **(D)** RF + HA drying.

By studying the influence of RF heat treatment on the microstructure of jujube slices after HAD (Figure 7), the microstructure of fresh jujube slices has a significant gap, regular arrangement, and honeycomb shape. Compared with the microstructure of dried jujube slices in different ways, the microstructure of dried jujube slices has different degrees of shrinkage, smaller gaps and even blocked, which is not suitable for drying. The reason for this phenomenon may be that with the increase in dryness, the components of the cell wall are gradually degraded, and the cell wall makes it difficult to play a certain supporting role, which leads to the gradual contraction and collapse of the water exchange channels between the micro skeleton and the cells and affects the microstructure morphology of jujube slices.

The microstructure of HAD jujube slices is small and closely arranged, the cell wall is seriously degraded, and the microscopic skeleton is seriously crumbled and collapsed. Compared with HAD, RF + HA heat treatment intervenes in the middle of drying, the microstructure gap of the jujube slices becomes more extensive, the arrangement is relatively loose, and the shape of the honeycomb is regular. The microstructure of the jujube slices is well maintained, and the drying time is relatively short. The reason for this phenomenon may be that the intervention of RF heat treatment heats from the inside, forming the same direction of water diffusion and heat transfer. The internal water quickly evaporated into water vapor, which had a certain expansion effect on the jujube slice cells and expanded the skeleton of the jujube slice microstructure, which could effectively reduce the damage to the microstructure of jujube slices caused by different heat transfer and mass transfer directions of single HAD, thus effectively reducing the difficulty of water diffusion. This is also similar to the conclusion of research on the microstructure changes of fruits and vegetables during drying (44), and the structural changes are also similar to the effect of temperature on persimmon (45).

### 3.2 Optimization analysis of orthogonal experiment

Through 9 groups of RF heat treatment, which measured *VC* content, a* and *DR* were applied to the HAD process test, and the analysis of the influence of different single factor tests on the quality of HAD slices was obtained (Table 5).

**Table 5:** Result analysis of HAD test for RF heat treatment.

No.		Factors			*VC* mg/100 g	a*	*DR* g/min
		A	B	C			
1		1	1	1	188.65	6.08	0.515
2		1	2	3	235.23	7.62	0.643
3		1	3	2	195.37	7.04	0.572
4		2	1	3	256.89	10.91	0.685
5		2	2	2	255.07	9.79	0.468
6		2	3	1	221.59	8.64	0.479
7		3	1	1	224.97	6.5	0.479
8		3	2	2	206.57	6.08	0.435
9		3	3	3	162.56	9.79	0.429
*VC* content	k1	0.206	0.224	0.212	Visual analysis of *VC* content		
	k2	0.245	0.232	0.219			
	k3	0.198	0.193	0.218			
	R	0.046	0.039	0.007			
a*	k1	6.913	7.637	7.073	Visual analysis of a*		
	k2	9.780	7.830	7.637			
	k3	7.457	8.490	9.440			
	R	2.867	0.853	2.367			
*DR*	k1	0.577	0.560	0.491	Visual analysis of *DR*		
	k2	0.544	0.515	0.492			
	k3	0.448	0.493	0.59			
	R	0.129	0.066	0.095			

According to the range analysis in Table 6, taking *VC* content as the index, the range of plate spacing is the largest, indicating that the order of influence on the drying effect of jujube slices is plate spacing > RF application time > RF interval time. Therefore, the parameter combination of the RF application time application mechanism under the HAD condition is A2B2C2.

**Table 6:** Experimental verification results of RF heat treatment in HAD.

Quality indicators	1	2	3	Average	Predicted value	RMSE	MAE
*VC* content (mg/g)	243.57	257.63	273.84	258.35	255.07	13.75	10.44
a*	8.53	10.56	9.32	9.47	9.79	0.906	0.833
*DR* (g/min)	0.64	0.65	0.629	0.64	0.64	0.00758	0.00734

Taking a* as the index, the range of plate spacing is the largest, and the primary and secondary order of influencing factors is plate spacing > RF interval time > RF application time. The parameter combination of the RF heat treatment application mechanism under HAD condition is A1B1C1.

Taking the *DR* as the index, the range of plate spacing is the largest, and the primary and secondary order of influencing factors is plate spacing > RF interval time > RF application time. The parameter combination of RF heat treatment application mechanism under HAD condition is A1B1C2.

#### 3.2.1 Comprehensive balance method was used to analyze the orthogonal test data of multiple indexes

The combined parameters of the RF heat treatment application mechanism obtained through the *VC* content, a*, and *DR* as indicators are inconsistent, and the comprehensive balance method needs to be further analyzed.

When the single factor condition selection index is plate spacing, the best plate spacing for the optimal value of *VC* content is 110 mm, and the best plate spacing for the optimal value of a* and *DR* is 100 mm. By comparing the difference between k1 and k2 of *VC* content index by 18.9%, the difference between k1 and k2 of a* by 29.3%, and the difference between k1 and k2 of *DR* by 6.06%, the optimal plate spacing was selected as 100 mm.

When the single factor condition selection index is RF application time, the application time of the optimal value of *VC* content is 3 min, and the best application time of color and *DR* is 2 min. The difference between k1 and k2 of the *VC* content index is 3.6%, but the difference between k1 and k2 of a* is 2.5%, and the difference between k1 and k2 of *DR* is 8.73%. Therefore, the best RF application time is 2 min.

When the single factor condition selection index is RF interval time, the best RF interval time for *VC* content and *DR* is 3 min, and the best RF interval time for a* is 2 min. By comparing the difference between k1 and k2 of *VC* content index and the difference between k2 and k2 of *DR* is 3.3 and 0.203%, but the difference between k1 and k2 of a* is 8.0%. Therefore, the best RF interval time is selected as 2 min; that is, the best parameter combination of the RF heat treatment application mechanism is A1B1C1.

#### 3.2.2 Matrix analysis method was used to analyze the orthogonal test data of multiple indexes

The index layer matrix *M*, factor layer matrix *F*, horizontal layer matrix *L* of *VC* content, a* and *DR,* and the weight matrix *E* of each index were established, respectively. The total weight matrix of the three indexes was *Y*, and the operation process was as follows in Table 7.

**Table 7:** Weight matrix analysis.

Layer matrix *M*	Actor layer matrix *F*	Horizontal layer matrix *L*	Weight matrix *W*	Total weight matrix of the indicators *Y*
$M1=\left[\begin{array}{ccc} 0.206 & 0 & 0\\ 0.245 & 0 & 0\\ 0.198 & 0 & 0\\ 0 & 0.224 & 0\\ 0 & 0.232 & 0\\ 0 & 0.193 & 0\\ 0 & 0 & 0.212\\ 0 & 0 & 0.219\\ 0 & 0 & 0.218 \end{array}\right]$	$F1=\left[\begin{array}{ccc} \frac{1}{0.649} & 0 & 0\\ 0 & \frac{1}{0.649} & 0\\ 0 & 0 & \frac{1}{0.649} \end{array}\right]$	$L1=\left[\begin{array}{c} \frac{0.046}{0.092}\\ \frac{0.039}{0.092}\\ \frac{0.007}{0.092} \end{array}\right]$	$E1=M1F1L1=\left[\begin{array}{c} 0.1585\\ 0.1598\\ 0.0232\\ 0.1725\\ 0.1513\\ 0.0226\\ 0.1630\\ 0.1429\\ 0.0256 \end{array}\right]$	$Y=1/3\left(E1+E2+E3\right)=\left[\begin{array}{c} 0.2523\\ 0.1956\\ 0.1377\\ 0.1519\\ 0.1599\\ 0.1301\\ 0.2347\\ 0.1589\\ 0.1593 \end{array}\right]=\left[\begin{array}{c} A1\\ A2\\ A3\\ B1\\ B2\\ B3\\ C1\\ C2\\ C3 \end{array}\right]$
$M2=\left[\begin{array}{ccc} -6.913 & 0 & 0\\ -9.78 & 0 & 0\\ -7.457 & 0 & 0\\ 0 & -7.637 & 0\\ 0 & -7.83 & 0\\ 0 & -8.49 & 0\\ 0 & 0 & -7.073\\ 0 & 0 & -7.637\\ 0 & 0 & -9.44 \end{array}\right]$	$F2=\left[\begin{array}{ccc} \frac{-1}{24.15} & 0 & 0\\ 0 & \frac{-1}{24.15} & 0\\ 0 & 0 & \frac{-1}{24.15} \end{array}\right]$	$L2=\left[\begin{array}{c} \frac{2.867}{6.087}\\ \frac{0.853}{6.087}\\ \frac{2.367}{6.087} \end{array}\right]$	$E2=M2F2L2=\left[\begin{array}{c} 0.1361\\ 0.0567\\ 0.12\\ 0.0149\\ 0.0454\\ 0.1369\\ 0.138\\ 0.0443\\ 0.152 \end{array}\right]$	
$M3=\left[\begin{array}{ccc} 0.577 & 0 & 0\\ 0.544 & 0 & 0\\ 0.448 & 0 & 0\\ 0 & 0.560 & 0\\ 0 & 0.515 & 0\\ 0 & 0.493 & 0\\ 0 & 0 & 0.491\\ 0 & 0 & 0.492\\ 0 & 0 & 0.590 \end{array}\right]$	$F3=\left[\begin{array}{ccc} \frac{1}{1.568} & 0 & 0\\ 0 & \frac{1}{1.568} & 0\\ 0 & 0 & \frac{1}{1.568} \end{array}\right]$	$L3=\left[\begin{array}{c} \frac{0.129}{0.291}\\ \frac{0.066}{0.291}\\ \frac{0.095}{0.291} \end{array}\right]$	$E3=M3F3L3=\left[\begin{array}{c} 0.163\\ 0.1538\\ 0.12678\\ 0.0809\\ 0.0743\\ 0.0712\\ 0.1022\\ 0.1024\\ 0.1227 \end{array}\right]$	

The total weight matrix *Y* is obtained through matrix calculation, and the value of *Y* is the weight corresponding to different factors at different levels. The order of the factors affecting *VC* content, a* and, *DR* of jujube slices is A > C > B. Therefore, the optimal process parameter combination is A1B2C1, that is, the plate spacing is 100 mm, the RF addition time is 3 min, and the RF heat treatment interval is 2 min.

Through the analysis and comparison of multi-index data of orthogonal design experiment by matrix analysis method and comprehensive balance method, the influencing factors are different in order, but the comprehensive balance method is similar to the formula scoring method (direct weight allocation) and has inevitable subjectivity, which is challenging to achieve objective analysis when multiple indicators cannot be assessed as the primary and secondary or all indicators are equally essential; The matrix analysis method has more advantages for the data processing of the orthogonal experiment with multiple indicators. Considering the objectivity and rigor of the experiment, the influence order is based on the data results of matrix analysis.

#### 3.2.3 Parameter combination verification test of the application mechanism of radiation heat treatment under HAD condition

The optimal parameter combination obtained by matrix analysis is A1B2C1 (*VC* content 255.07 mg/g, a* −9.79, *DR* (0.64 g/min)). A process verification test is required for the optimal combination parameters of the orthogonal test. The hot air temperature is 65°C, the wind speed is 0.5 m/s, the plate spacing is 100 mm, and the RF application time is 3 min. Under the condition of a 2 min RF interval, three groups of parallel tests were carried out, and the results were shown in Table 6. The *VC* content of the jujube slices measured by the best parameter combination of the orthogonal test was 258.35 mg/g, a* was −9.47, and the *DR* was 0.64 g/min. The error analysis of the experimental results showed that the root mean square errors (RMSE) of the three quality indexes were 13.75, 0.906, and 0.00758, respectively, indicating that the quality indexes of winter jujube slices reached an optimal level under the condition of the best parameter combination as A1B2C1. When all the errors are equally essential, the mean absolute error (MAE) gives equal weight to the three indexes, and the verification results are 10.44, 0.833, and 0.00734, respectively, indicating that the optimized process parameters can obtain better drying quality of winter jujube slices by RF + HA.

## 4 Conclusion

The aim of this study was to investigate the factors that influence RF + HA on the quality and texture properties of winter jujube slices. We found that in the orthogonal test, taking *VC* content, a*, value and *DR* as indicators, the range of plate spacing was more significant than the other two factors; that is, plate spacing had the most significant influence on the three evaluation indicators of winter jujube slices. The comprehensive balance method and matrix analysis method were used to optimize the orthogonal experimental results; considering the science and objectivity of multi-index analysis, the primary and secondary order of influence on the index is plate spacing > RF heat treatment interval time > RF heat treatment application time. Compared with untreated fresh winter jujube slices, the color of the optimized process was red and the color quality was better. RF + HA has better texture characteristics, the jujube slices were arranged loosely, and the honeycomb shape was regular. The results obtained by the comprehensive balance method and matrix analysis method are helpful in better understanding the drying process of winter jujube slices in the RF + HA and provide helpful information for the selection of drying methods in the food processing industry for winter jujube slices. In addition, by analyzing the influence of different process parameters on the quality characteristics of winter jujube slices, the best drying conditions were determined, which is of great value for obtaining products with high nutritional quality and improving the process added value of winter jujube slices.

## Data Availability

The original contributions presented in the study are included in the article/Supplementary material, further inquiries can be directed to the corresponding author.
